# Unravelling the significance of Fecal Micro RNA Profile in Alzheimer's Disease

**DOI:** 10.1002/alz70855_098838

**Published:** 2025-12-23

**Authors:** Kavisha Sandaru Katuwawala, Warnakulasuriya Mary Ann Dipika Binosha Fernando, Prashant Bharadwaj, Ralph N Martins

**Affiliations:** ^1^ EDITH COWAN UNIVVERSITY AUSTRALIA, Perth, Western Australia, Australia; ^2^ Centre of Excellence for Alzheimer's Disease Research and Care, School of Medical and Health Sciences, Edith Cowan University, Joondalup, Western Australia, Australia; ^3^ Edith Cowan University, Perth, Australia; ^4^ Edith Cowan University, Perth, Western Australia, Australia; ^5^ School of Medical and Health Sciences, Edith Cowan University, Perth, Western Australia, Australia

## Abstract

**Background:**

Alzheimer's Disease (AD) is a complex neurodegenerative disorder characterized by cognitive decline and memory loss. Emerging research suggests that gut microbiota play a significant role in AD progression through mechanisms like neuroinflammation and neurotransmitter dysregulation. Fecal microRNAs (miRNAs) have gained attention as non‐invasive biomarkers reflecting gut‐brain communication, offering potential insights into disease pathogenesis and therapeutic targets. This review examines the significance of fecal miRNA profiles in AD, focusing on their role in early diagnosis, disease monitoring, and potential therapeutic intervention.

**Method:**

A systematic literature search was conducted using PubMed, Scopus, Web of Science, and Google Scholar for studies published from 2010 to 2023. Inclusion criteria were based on research articles investigating fecal miRNA expression in AD, miRNAs involved in gut‐brain communication, and studies highlighting miRNAs as diagnostic or prognostic biomarkers. Search terms such as “Alzheimer's Disease,” “fecal microRNA,” “gut microbiome,” “biomarkers,” and “gut‐brain axis” were used. A total of 40 studies, including both clinical and preclinical research, met the inclusion criteria and were reviewed.

**Result:**

Key miRNAs such as miR‐146a, miR‐155, and miR‐34a were consistently dysregulated, indicating their involvement in neuroinflammatory pathways and synaptic dysfunction. These miRNAs also influenced amyloid precursor protein (APP) processing and tau phosphorylation, critical factors in AD pathogenesis. Additionally, miR‐132 and miR‐181c were associated with cognitive decline and AD severity, suggesting their potential as non‐invasive biomarkers for disease progression. Preclinical studies also demonstrated that dietary interventions and probiotics could modulate fecal miRNA expression, indicating potential therapeutic strategies targeting the gut microbiome.

**Conclusion:**

Fecal miRNA profiles offer valuable insights into the gut‐brain axis in Alzheimer's Disease and serve as promising non‐invasive biomarkers for early diagnosis and disease monitoring. Altered miRNA expression reflects gut dysbiosis and neuroinflammatory responses, making them potential targets for therapeutic interventions. Future research should focus on validating these findings through large‐scale clinical trials and exploring how dietary and probiotic treatments can modify miRNA expression to benefit AD patients. This review emphasizes the need for a multidisciplinary approach to better understand the role of the gut microbiome in neurodegenerative diseases and develop novel strategies for AD management

